# Supplementary material for: EnzML: multi-label prediction of enzyme classes using InterPro signatures
Source: BMC Bioinformatics. 2012 Apr 25;13:61. doi: 10.1186/1471-2105-13-61 (PMC3483700; doi:10.1186/1471-2105-13-61)
Supplement: Addtional file 5 — The Java code to format the data files, evaluate and predict. The file enzml_java_code.tar.gz contains the Java code used to format database data to ARFF and XML formats, to execute cross and train-test (jackknife) evaluations and to record evaluation results to database. More information is included in the readme.txt file and the Javadoc files. The code can be used with a MySQL database. To use a different database software, other JDBC drivers might be required. [file 1471-2105-13-61-S5.gz › java_code/enzml2011/doc/uk/ac/ed/inf/taweka/mulan/MulanDataSetWriter.html]

MulanDataSetWriter


---


|  |  |  |
| --- | --- | --- |
| **Overview** \| **Package** \| **Class** \| **Use** \| **Tree** \| **Deprecated** \| **Index** \| **Help** | |  |
| **PREV CLASS**   **NEXT CLASS** | **FRAMES**    **NO FRAMES**     **All Classes** |  |
| SUMMARY: NESTED \| FIELD \| CONSTR \| METHOD | DETAIL: FIELD \| CONSTR \| METHOD |  |


---


## uk.ac.ed.inf.taweka.mulan Class MulanDataSetWriter

```
java.lang.Object
  uk.ac.ed.inf.taweka.DataSetWriter
      uk.ac.ed.inf.taweka.mulan.MulanDataSetWriter
```

---

``` public class MulanDataSetWriter extends DataSetWriter ```

This class generates a data set (instances, attributes, classes) in Mulan
style (for multi-label machine learning). The main difference compared with
the classic Weka ARFF, is that there are multiple classes and each is simply
listed as a normal binary {0, 1} attribute. The class attributes must also be
specified in a separate Mulan XML.

**Version:**
:   7 May 2010

**Author:**
:   Luna De Ferrari luna.deferrari-at-ed.ac.uk

---

**Field Summary**

**Fields inherited from class uk.ac.ed.inf.taweka.DataSetWriter**

`DUMMY_VALUE`


**Constructor Summary**

`MulanDataSetWriter(ArffGenerator arffGenerator,
uk.ac.ed.inf.utils.OneToManyMap instanceAttributeMap,
uk.ac.ed.inf.utils.OneToManyMap instanceClassMap)`
  
          The set of class attributes.


**Method Summary**

**Methods inherited from class uk.ac.ed.inf.taweka.DataSetWriter**

`generateDataSet, getArffProperties, getAttributeFactory, getAttributes, getClassName, getDataSetName, getInstanceAttributeData, getInstanceClassData, getInstances, getLog, getProperties, writeInstancesToFile`

**Methods inherited from class java.lang.Object**

`equals, getClass, hashCode, notify, notifyAll, toString, wait, wait, wait`

**Constructor Detail**

### MulanDataSetWriter

```
public MulanDataSetWriter(ArffGenerator arffGenerator,
                          uk.ac.ed.inf.utils.OneToManyMap instanceAttributeMap,
                          uk.ac.ed.inf.utils.OneToManyMap instanceClassMap)
```

:   The set of class attributes. Mulan multi-label learning can accept
    multiple class attributes formatted as normal attributes. The Mulan XML
    file contains the attributes to be used as class attributes.


---


|  |  |  |
| --- | --- | --- |
| **Overview** \| **Package** \| **Class** \| **Use** \| **Tree** \| **Deprecated** \| **Index** \| **Help** | |  |
| **PREV CLASS**   **NEXT CLASS** | **FRAMES**    **NO FRAMES**     **All Classes** |  |
| SUMMARY: NESTED \| FIELD \| CONSTR \| METHOD | DETAIL: FIELD \| CONSTR \| METHOD |  |


---
